# Homozygous of *MRP4* Gene rs1751034 C Allele Is Related to Increased Risk of Intravenous Immunoglobulin Resistance in Kawasaki Disease

**DOI:** 10.3389/fgene.2021.510350

**Published:** 2021-03-15

**Authors:** Yanfei Wang, Yufen Xu, Ping Huang, Di Che, Zhouping Wang, Xijing Huang, Xiaofei Xie, Wei Li, Li Zhang, Xiaoqiong Gu

**Affiliations:** ^1^Department of Pediatric Cardiology, Guangzhou Women and Children’s Medical Center, Guangzhou Medical University, Guangzhou, China; ^2^Department of Blood Transfusion and Clinical Biological Resource Bank, Institute of Pediatrics, Guangzhou Women and Children’s Medical Center, Guangzhou Medical University, Guangzhou, China

**Keywords:** coronary artery lesion, rs1751034, multi-drug resistance protein 4, intravenous immunoglobulin resistance, Kawasaki disease

## Abstract

**Background:** Kawasaki disease (KD) is a systemic vasculitis in childhood, which mainly causes damage to coronary arteries, and intravenous immunoglobulin (IVIG) is the initial therapy. IVIG resistance increased risk of coronary complication in KD. And genetic background is involved in the occurrence of IVIG resistance. Our previous study indicated the susceptibility of *Multi-drug resistance protein 4 (MRP4)* SNPs to KD. This study was to clarify the relationship between *MRP4* polymorphisms and IVIG resistance.

**Methods:** We genotyped the six polymorphisms of *MRP4* gene in 760 cases of KD using Taqman methods.

**Results:** Among the six polymorphisms, only the rs1751034 polymorphism was significantly associated with IVIG resistance in KD [CC vs. TT: adjusted odds ratio (OR) = 2.54, 95% confidence interval (CI) = 1.21–5.34; CC vs. TT/TC: adjusted OR = 2.33, 95% CI = 1.12–4.83, *p* = 0.023]. Combined analysis of three polymorphisms indicated that patients with 3–6 risk genotypes exhibited significantly elevated risk of IVIG resistance, when compared with those with 0–2 risk genotypes (adjusted OR = 1.52, 95% CI = 1.04–2.22, *p* = 0.0295). Stratified analysis revealed that in term of age and gender, rs1751034 CC carriers were associated with increased risk of IVIG resistance in those aged ≤ 60 months (adjusted OR = 2.65, 95% CI = 1.23–5.71, *p* = 0.0133). The presence of three or more risk genotypes was significantly associated with risk of IVIG resistance in children younger than 5 years of age and males.

**Conclusion:** Our results suggest that *MRP4* rs1751034 CC is associated with increased risk of IVIG resistance in KD.

## Introduction

Kawasaki disease (KD), the most common cause of acquired heart disease in childhood in developed countries, is an acute febrile illness which predominantly affects children younger than 5 years of age and leads to coronary artery complications (Group, 2014; McCrindle et al., 2017). However, timely initiation of treatment with intravenous immunoglobulin (IVIG) has reduced the incidence of coronary artery aneurysms (CAAs) from 25 to 4% (McCrindle et al., 2017; de Graeff et al., 2018). Many studies have discussed the dosing and timing of IVIG. The American Heart Association (AHA) recommends a single infusion of 2 g/kg preferably given during the first 10 days of illness (McCrindle et al., 2017). Unfortunately, approximately 10–20% of patients with KD develops recrudescent or persistent fever at least 36 h after the end of their primary therapy with IVIG infusion and are termed IVIG resistance (McCrindle et al., 2017). Many studies have shown that patients who are resistant to initial IVIG are at increased risk of developing coronary artery abnormalities (Wei et al., 2015; de Graeff et al., 2018). Thus, efforts such as scoring systems and risk prediction models have been constructed to identify patients likely to be resistant to IVIG (Sleeper et al., 2011; Fu et al., 2013; Hua et al., 2017). There may be varying mechanisms depending on the condition being treated with IVIG in KD. IVIG contains antibodies and possesses effects on platelet adhesion, oxidative stress and neutrophil function, and also inhibits T cell activation, tumor necrosis factor –α production, and matrix metalloproteinase-9 activity, etc. (Lo and Newburger, 2018). However, the genetic basis of IVIG resistance is unknown. Researchers suggest that response to IVIG is influenced by variants in several different genes (Kuo et al., 2013, 2015).

Recent studies show that multi-drug resistance protein 4 (MRP4), also known as ATP-binding cassette, sub-family C, member 4 (ABCC4), plays a role in cAMP homeostasis, a key-pathway in vascular biology and in platelet functions (Belleville-Rolland et al., 2016). Based on genome-wide family linkage and association mapping, Khor et al. (2011) concluded that MRP4 could play a fundamental role in KD pathogenesis with effects on immune activation and vascular response to injury. Che et al. (2018b) indicated that specific single-nucleotide polymorphism (SNP) in the *MRP4* gene may increase susceptibility to KD. All these results suggest that MRP4 may exert a vital role in KD. However, there is no report on relationship of MRP4 and IVIG response yet. In this study, we aimed to evaluate the association between *MRP4* gene polymorphisms and IVIG resistance in KD in a southern population of China.

## Materials And Methods

### Study Population

A total of 760 patients who had been diagnosed with KD were recruited from Guangzhou Women and Children’s Medical Center between January 2012 and December 2017. The diagnosis of KD was made according to the statement of AHA (Newburger et al., 2004). All patients received a single dose of 2 g/kg IVIG at the diagnosis of KD. IVIG resistance is defined as persistent or recrudescent fever at least 36 h and <7 days after completion of first IVIG infusion (McCrindle et al., 2017). About 2 ml of whole blood was collected from each participant for genomic DNA extraction (Qiagen, Dusseldorf, Germany). Written informed consent was obtained from the guardians of participants. This study was conducted with the approval form the Guangzhou Women and Children’s Medical Center Ethics Committee (2014073009).

### Polymorphism Selection and Genotyping

The interested polymorphisms of *MRP4* were selected according to the previously described standards (Che et al., 2018b,c). In total, six SNPs in the *MRP4* gene were analyzed (rs1751034, rs3742106, rs9561778, rs3765534, rs868853, and rs7320375). The first five SNPs were identified in a study associated with susceptibility to KD based on a population of South China (Che et al., 2018b). The last SNP, an intron variant within *MRP4*, retained evidence of association with KD in a genome-wide association study (Khor et al., 2011).

Total genomic DNA was extracted from the blood sample using a TIANamp Blood DNA Kit (centrifugal column; Tiangen, Beijing, China) according to the manufacturer’s instructions. Genotyping was conducted using TaqMan technology as previously described (Che et al., 2018a; He et al., 2018). Briefly, high-quality genomic DNA samples were genotyped by real-time PCR assay using multiple gene-specific primer pairs on a 7900 HT sequence detector system (Applied Biosystems, Foster City, CA, United States). Eight positive controls used for comparisons and eight negative controls used for accuracy were included in each 384-well plate. Additionally, 10% samples were randomly selected to be performed in duplicate to test the reproducibility.

### Statistical Analysis

The genotype frequencies of each SNP as well as the demographic variables (e.g., age and gender) between cases with IVIG resistance and IVIG sensitivity were compared by using the *χ*^2^ test. Stratified analysis was performed by age and gender. Odds ratio (OR) and corresponding 95% confidence interval (CI), which were applied to analyze the association between SNPs and IVIG response, were calculated by unconditional logistic regression analyses adjusted for age and gender. Statistical analyses were performed by using SAS software (version 9.3; SAS Institute, Cary, NC, United States). All values of *p* in the current study were two-sided, and a value of *p* less than 0.05 was considered statistically significant.

## Results

### Characteristics of the Population

Totally, 760 patients were enrolled in our study. The demographics of the participants are shown in Table 1. There were 148 patients identified as IVIG resistance (IVIG-R) and 612 cases as IVIG sensitivity (IVIG-S). The average age was 29.02 months (ranging from 1 to 125 months) in IVIG-R group and 27.9 months (ranging from 1 to 156 months) in IVIG-S group. The IVIG-R group comprised 97 (65.54%) male patients and 51 (34.46%) female patients, and the IVIG-S group included 417 (68.14%) males and 195 (31.86%) females. No significant difference was observed in age (*p* = 0.0656) or gender (*p* = 0.5462) between patients of IVIG-R and IVIG-S.

**Table 1:** Frequency distribution of selected characteristics in Kawasaki disease (KD) cases.

Variables	IVIG-R^a^ (*n* = 148)		IVIG-S^b^ (*n* = 612)		*p*^c^
	n	%	n	%	
Age range, month	1–125		1–156		0.0656
Mean ± SD	29.02 ± 25.37		27.90 ± 23.11		
≤60	129	87.16	564	92.16	
>60	19	12.84	48	7.84	
Gender					0.5462
Male	97	65.54	417	68.14	
Female	51	34.46	195	31.86	

a Kawasaki disease patients who were resistant to IVIG therapy.

b Kawasaki disease patients who were sensitive to IVIG therapy.

c Two-sided *χ*^2^ test for distributions between Kawasaki disease patients with IVIG resistance and IVIG sensitivity.

### Association Between *MRP4* Gene Polymorphisms and IVIG-R in KD

To explore the relationship between *MRP4* gene polymorphisms and IVIG-R in KD, we detected the genotype frequency distributions of IVIG-R and IVIG-S patients. As shown in Table 2, among the six investigated SNPs, significant difference was found in the genotype distributions for rs1751034 T/C (*p* = 0.0441) between IVIG-R and IVIG-S patients. After adjusting for age and gender, when the rs1751034 TT genotype was used as the reference, the CC variant genotype was associated with an increased risk of IVIG-R (adjusted OR = 2.54, 95% CI = 1.21–5.34, *p* = 0.0143 for CC vs. TT; adjusted OR = 2.33, 95% CI = 1.12–4.83, *p* = 0.023 for CC vs. TT/TC). However, the other five SNPs were not associated with IVIG-R. A combination analysis showed that the risk of IVIG-R in KD was significantly higher in patients with 3–6 risk genotypes than in those with 0–2 risk genotypes (adjusted OR = 1.52, 95% CI = 1.04–2.22, *p* = 0.0295).

**Table 2:** Genotype frequency distribution of MRP4 in KD cases.

Genotype	IVIG-R (*n* = 148)	IVIG-S (*n* = 612)	*p*^a^	OR (95% CI)	*p*	Adjusted OR (95% CI)	Adjusted *p*^b^
**rs1751034 T/C**							
TT	82 (55.41)	387 (63.24)	**0.0441**	1.00		1.00	
TC	54 (36.49)	203 (33.17)		1.26 (0.86–1.84)	0.2446	1.26 (0.86–1.84)	0.2427
CC	12 (8.11)	22 (3.59)		2.57 (1.23–5.41)	**0.0126**	2.54 (1.21–5.34)	**0.0143**
Dominant	66 (44.59)	225 (36.76)	0.0807	1.38 (0.96–1.99)	0.0793	1.38 (0.96–1.99)	0.0803
Recessive	136 (91.89)	590 (96.41)	**0.0268**	2.37 (1.14–4.90)	**0.0203**	2.33 (1.12–4.83)	**0.0230**
**rs3742106 A/C**							
AA	30 (20.27)	139 (22.71)	0.4010	1.00		1.00	
AC	80 (54.05)	293 (47.88)		1.27 (0.79–2.02)	0.3223	1.27 (0.80–2.02)	0.3190
CC	38 (25.68)	180 (29.41)		0.98 (0.58–1.66)	0.9346	0.97 (0.57–1.65)	0.9215
Dominant	118 (79.73)	473 (77.29)	0.5179	1.16 (0.74–1.80)	0.5217	1.16 (0.74–1.80)	0.5236
Recessive	110 (74.32)	432 (70.59)	0.3631	0.83 (0.55–1.25)	0.3676	0.83 (0.55–1.24)	0.3551
**rs9561778 G/T**							
GG	70 (47.30)	307 (50.16)	0.8199	1.00		1.00	
GT	67 (45.27)	261 (42.65)		1.13 (0.78–1.64)	0.5339	1.12 (0.77–1.63)	0.5494
TT	11 (7.43)	44 (7.19)		1.10 (0.54–2.23)	0.7994	1.08 (0.53–2.20)	0.8301
Dominant	78 (47.30)	307 (50.16)	0.5313	1.12 (0.78–1.61)	0.5316	1.11 (0.78–1.60)	0.5529
Recessive	137 (92.57)	568 (92.81)	0.9187	1.04 (0.52–2.06)	0.9177	1.02 (0.51–2.04)	0.9478
**rs3765534 C/T**							
CC	131 (88.51)	556 (90.85)	0.4931	1.00		1.00	
CT	16 (10.81)	55 (8.99)		1.24 (0.69–2.22)	0.4825	1.26 (0.70–2.28)	0.4398
TT	1 (0.68)	1 (0.16)		4.24 (0.26–68.30)	0.3078	4.64 (0.29–75.21)	0.2801
Dominant	17 (11.49)	56 (9.15)	0.3964	1.29 (0.73–2.29)	0.3877	1.32 (0.74–2.35)	0.3488
Recessive	147 (99.32)	611 (99.84)	0.3333	4.16 (0.26–66.84)	0.3148	4.51 (0.28–72.97)	0.2892
**rs868853 T/C**							
TT	107 (72.30)	417 (68.14)	0.6126	1.00		1.00	
TC	38 (25.68)	181 (29.58)		0.82 (0.54–1.23)	0.3365	0.82 (0.55–1.24)	0.3486
CC	3 (2.03)	14 (2.29)		0.84 (0.24–2.96)	0.7801	0.82 (0.23–2.91)	0.7571
Dominant	41 (27.70)	195 (31.86)	0.3224	0.82 (0.55–1.22)	0.3269	0.82 (0.55–1.22)	0.3345
Recessive	145 (97.97)	598 (97.71)	0.8456	0.88 (0.25–3.12)	0.8476	0.87 (0.25–3.05)	0.8213
**rs7320375 A/G**							
AA	97 (65.54)	382 (62.42)	0.5861	1.00		1.00	
AG	43 (29.05)	203 (33.17)		0.83 (0.56–1.24)	0.3713	0.84 (0.56–1.25)	0.3840
GG	8 (5.41)	27 (4.41)		1.17 (0.51–2.65)	0.7121	1.18 (0.52–2.67)	0.7004
Dominant	51 (34.46)	230 (37.58)	0.4785	0.87 (0.60–1.27)	0.4803	0.88 (0.60–1.28)	0.4959
Recessive	140 (94.59)	585 (95.59)	0.6118	1.24 (0.55–2.78)	0.6053	1.25 (0.55–2.81)	0.5949
**Combined effect of risk genotypes**							
0–2	84 (56.76)	409 (66.83)	**0.0227**	1.00		1.00	
3–6	64 (43.24)	203 (33.17)		1.52 (1.05–2.22)	**0.0283**	1.52 (1.04–2.22)	**0.0295**

a Two-sided *χ*^2^ test for distributions between Kawasaki disease patients with IVIG resistance and IVIG sensitivity.

b Adjusted for age and gender status in logistic regress models.

Bold values indicate value of p less than 0.05.

### Stratification Analysis

We further explored the association between the genotypes of six selected SNPs in the *MRP4* gene and the risks of IVIG-R by stratifying the patients by age and gender (Table 3). When the patients were stratified by age after adjusting for gender, the CC genotype of the rs1751034 T > C polymorphism contributed to a higher occurrence of IVIG-R compared with the TT/TC genotypes in patients aged≤60 months (adjusted OR = 2.65, 95% CI = 1.23–5.71, *p* = 0.0133), but not in patients aged >60 months. When the 6 risk genotypes were combined into a new variable, compared with those with 0–2 risk genotypes, patients with 3–6 risk genotypes had a higher risk in those aged≤60 months (adjusted OR = 1.86, 95% CI = 1.26–2.74, *p* = 0.0018) and males (adjusted OR = 1.62, 95% CI = 1.03–2.55, *p* = 0.0354). There was no significant association with other stratified analyses.

**Table 3:** Stratification analysis of susceptibility in KD patients.

Variables	rs1751034 (IVIG-R/IVIG-S)		*p*	OR (95% CI)	*p*^a^	Adjusted OR (95% CI)	Adjusted *p*^b^	combined analysis (IVIG-R/IVIG-S)		*p*	OR (95% CI)	*p*^a^	Adjusted OR (95% CI)	Adjusted *p*^b^
	TT/TC	CC						0–2	3–6					
**Age, months**														
≤60	118/545	11/19	**0.0172**	2.67 (1.24–5.77)	**0.0122**	2.65 (1.23–5.71)	**0.0133**	69/384	60/180	**0.0020**	1.86 (1.26–2.74)	**0.0018**	1.86 (1.26–2.74)	**0.0018**
>60	18/45	1/3	0.8764	0.83 (0.08–8.56)	0.8780	0.87 (0.08–8.96)	0.9036	15/25	4/23	0.0375	0.29 (0.08–1.00)	0.0503	0.29 (0.08–1.01)	0.0509
**Gender**														
Male	90/404	7/13	0.0821	2.42 (0.94–6.23)	0.0677	2.35 (0.91–6.08)	0.0775	54/279	43/138	**0.0392**	1.61 (1.03–2.52)	**0.0378**	1.62 (1.03–2.55)	**0.0354**
Female	46/186	5/9	0.1816	2.25 (0.72–7.02)	0.1640	2.32 (0.74–7.31)	0.1517	30/130	21/65	0.2999	1.40 (0.74–2.63)	0.2969	1.47 (0.78–2.78)	0.2373

a Two-sided *χ*^2^ test for distributions between Kawasaki disease patients with IVIG resistance and IVIG sensitivity.

b Adjusted for age and gender, stratified by gender (adjusted for age), and stratified by age (adjusted for gender).

Bold values indicate value of p less than 0.05.

## Discussion

Although KD is not a genetic disease, evidence for a genetic component to KD susceptibility has been established in many studies (Group, 2014; McCrindle et al., 2017). Family linkage studies and genome-wide disequilibrium analyses have implicated SNPs in susceptibility to KD in different genes (McCrindle et al., 2017). While only few studies have assessed the genetic relationship between KD and IVIG non-responsiveness (Weng et al., 2010; Shrestha et al., 2011, 2012; Portman et al., 2013; Shendre et al., 2014; Huang et al., 2016; Ahn et al., 2018), including IFN-gamma, DC-SIGN, IL-1B, FcγR, BAZ1A, STX1B, high mobility group box 1 (HMGB1), etc. It is likely that host genetic factors, such as polymorphisms in the immune and inflammatory pathways, play a role in both the response and resistance to IVIG.

Members of the ATP-binding cassette transporter superfamily are widely recognized as major contributors to controlling drug distribution and pharmacokinetics and the acquisition of anticancer drug resistance (Massimi et al., 2015). MRP4 (ABCC4) is a member of the C subfamily of ATP-binding cassette transporters (Russel et al., 2008). It is expressed in a variety of tissues and cells, including endothelium and platelet, which play a vital role in KD (Ritter et al., 2005). In the present study, we investigated the association between the *MRP4* gene polymorphisms and IVIG resistance in 760 KD patients. Our study showed that only the *MRP4* gene rs1751034 CC variant genotype was correlated to an increased risk to IVIG resistance in KD patients younger than 5 years of age. Patients with 3–6 risk genotypes had significantly higher risk of IVIG resistance in KD than those with 0–2 risk genotypes, especially in children aged ≤60 months and males. This is the first study in which *MRP4* gene SNPs were found to be associated with IVIG resistance in KD. Che et al. (2018b) observed six SNPs of *MRP4* gene (rs7986087, rs3742106, rs9561778, rs3765534, rs1751034, and rs868853) in KD in a southern Chinese population and indicated that the rs7986087 T variant genotype was associated with higher susceptibility, while the rs868853 T variant genotype was associated with lower susceptibility. Khor et al. (2011) observed three SNPs of *MRP4* gene (rs7986087, rs7320375, and rs7329490) and noted that the above three *MRP4* SNPs were only found in KD cases, indicating the risk of developing KD.

The remarkable characteristic of MRP4 is its ability to transport arrange of endogenous molecules that possess a key role in cellular communication and signaling, including cyclic nucleotides (cAMP and cGMP), ADP, prostaglandins, urate and conjugated steroid and glutathione (Ritter et al., 2005; Russel et al., 2008). In particular, MRP4 mediates the efflux of prostaglandin E1 (PGE1) and prostaglandin E2 (PGE2), a function that is inhibited by non-steroidal anti-inflammatory agents *in vitro* (Reid et al., 2003). And IVIG acted as a trigger for PGE2 expression in the acute stage of KD and a change of plasma PGE2 levels may be related to IVIG resistance (Kuo et al., 2016).

The present study presented that carriers of C allele in homozygous at rs1751034 of *MRP4* gene showed a significant association with IVIG resistance in KD. *MRP4* gene rs1751034 was involved in some diseases and the metabolism of anti-virus drugs. In HIV-positive patients, *MRP4* rs1751034 could be a genetic determinant of kidney tubular dysfunction (Salvaggio et al., 2017). The influence of transporters on the kinetics of efavirenz was also proved with significant correlations between the pharmacokinetic parameters of efavirenz and *MRP4* (rs1751034 and rs2274407) in HIV infected patients (Sanchez-Martin et al., 2016).

The variant of *MRP4* and the expression of the gene were related to drug sensitivity and resistance in various diseases. Ban et al. (2010) reported that *MRP4* G2269A was associated to high concentration of 6-thioguanine nucleotide and might be a new factor accounting for thiopurine sensitivity in Japanese patients with inflammatory bowel disease. Patients with any of the three *MRP4* homozygous variant allele (G2269A, C912A, and G559T) required high frequency of 6-mercaptopurine dose reduction compared with non-homozygous individuals and *MRP4* genotyping may be useful for personalizing the therapeutic dose of 6-mercaptopurine during the acute lymphoblastic leukemia maintenance therapy in Japanese (Tanaka et al., 2015). The results of Massimi et al. (2015) suggested exposing cells to low nontoxic aspirin dosages can induce *MRP4* gene expression alterations that may lead to the efflux transporter protein overexpression, thus increasing cellular detoxification of aspirin. Platelet MRP4 overexpression induced by aspirin treatment has a role in reducing aspirin action, and platelets that present high MRP4 levels have an increase of residual platelet reactivity in patients under chronic aspirin treatment (Massimi et al., 2016), indicating that MRP4 upregulation may be a pivotal aspect of aspirin resistance. Tsukamoto et al. (2019) focused on one non- synonymous SNP variant of *MRP4* (rs11568658, 559 G > T, G187W) and found that the substitution of Gly at position 187 of MRP4 to Trp resulted in reduced SN-38 resistance. The rs1751034 SNP (T > C) in the present study is a variant which do not change the structure of target protein. The reason why *MRP4* SNP is related to IVIG resistance in KD is still unknown. Whether *MRP4* SNPs affect the expression or function of transporter protein, leading to different transportation of immunoglobulin or other molecules, therefore, IVIG resistance appeared in KD patients, needs further studies and the expected results may lead to greater therapeutic efficacy in the treatment of KD.

Although *MRP4* gene polymorphisms were discovered to be associated with IVIG resistance in KD in this study, there is limitation that the current samples are recruited from a single center and mostly based on a southern Chinese population. Population from multicenter and multiple genetic backgrounds are required to validate the relationship between *MRP4* and KD.

## Conclusion

The present study displayed that the homozygous of *MRP4* gene rs1751034 C allele is significantly associated with IVIG resistance in KD, especially in patients younger than 5 years old and boys. Researches into the functions of MRP4 in KD are useful in the individualization of IVIG therapy and will improve the therapeutic effect in IVIG-unresponsive KD. Further studies are needed to verify the *MRP4* SNPs in IVIG resistance and explore the underlying mechanisms of MRP4 in KD.

## Data Availability Statement

The raw data supporting the conclusions of this paper will be made available by the authors, without undue reservation, to any qualified researcher.

## Ethics Statement

The studies involving human participants were reviewed and approved by Guangzhou Women and Children’s Medical Center Ethics Committee. Written informed consent to participate in this study was provided by the participants’ legal guardian/next of kin.

## Author Contributions

LZ and XG designed and supervised the study. YW, YX, PH, and DC performed the study and analyzed the data. YW wrote the manuscript. ZW, XX, and WL collected the samples and information. All authors contributed to the article and approved the submitted version.

### Conflict of Interest

The authors declare that the research was conducted in the absence of any commercial or financial relationships that could be construed as a potential conflict of interest.

## Abbreviations

KD: Kawasaki disease
IVIG: Intravenous immunoglobulin
CAA: Coronary artery aneurysm
MRP4: Multi-drug resistance protein 4
SNP: Single nucleotide polymorphism
OR: Odds ratio
CI: Confidence interval
ABCC4: Adenosine triphosphate (ATP)-binding cassette, sub-family C, member 4
PGE: Prostaglandin E.
